# Impacts of Surface Water Diversions for Marijuana Cultivation on Aquatic Habitat in Four Northwestern California Watersheds

**DOI:** 10.1371/journal.pone.0120016

**Published:** 2015-03-18

**Authors:** Scott Bauer, Jennifer Olson, Adam Cockrill, Michael van Hattem, Linda Miller, Margaret Tauzer, Gordon Leppig

**Affiliations:** 1 California Department of Fish and Wildlife, Eureka, California, United States of America; 2 National Marine Fisheries Service, Arcata, California, United States of America; The Ohio State University, UNITED STATES

## Abstract

Marijuana (*Cannabis sativa* L.) cultivation has proliferated in northwestern California since at least the mid-1990s. The environmental impacts associated with marijuana cultivation appear substantial, yet have been difficult to quantify, in part because cultivation is clandestine and often occurs on private property. To evaluate the impacts of water diversions at a watershed scale, we interpreted high-resolution aerial imagery to estimate the number of marijuana plants being cultivated in four watersheds in northwestern California, USA. Low-altitude aircraft flights and search warrants executed with law enforcement at cultivation sites in the region helped to validate assumptions used in aerial imagery interpretation. We estimated the water demand of marijuana irrigation and the potential effects water diversions could have on stream flow in the study watersheds. Our results indicate that water demand for marijuana cultivation has the potential to divert substantial portions of streamflow in the study watersheds, with an estimated flow reduction of up to 23% of the annual seven-day low flow in the least impacted of the study watersheds. Estimates from the other study watersheds indicate that water demand for marijuana cultivation exceeds streamflow during the low-flow period. In the most impacted study watersheds, diminished streamflow is likely to have lethal or sub-lethal effects on state-and federally-listed salmon and steelhead trout and to cause further decline of sensitive amphibian species.

## Introduction

Marijuana has been cultivated in the backwoods and backyards of northern California at least since the countercultural movement of the 1960s with few documented environmental impacts [1]. Recent increases in the number and size of marijuana cultivation sites (MCSs) appear to be, in part, a response to ballot Proposition 215, the Compassionate Use Act (1996). This California law provides for the legal use and cultivation of medical marijuana. In 2003, legislation was passed in an attempt to limit the amount of medical marijuana a patient can possess or cultivate (California State Senate Bill 420). However, this legislation was struck down by a 2010 California Supreme Court decision (*People v*. *Kelly*). As a result of Proposition 215 and the subsequent Supreme Court ruling, the widespread and largely unregulated cultivation of marijuana has increased rapidly since the mid-1990s in remote forested areas throughout California [2]. California is consistently ranked highest of all states for the number of outdoor marijuana plants eradicated by law enforcement: from 2008–2012 the total number of outdoor marijuana plants eradicated in California has ranged from 53% to 74% of the total plants eradicated in the United States [3]. In spite of state-wide prevalence, there is not yet a clear regulatory framework for the cultivation of marijuana, and from an economic viewpoint there is little distinction between plants grown for the black market and those grown for legitimate medical use [4].

Northwestern California has been viewed as an ideal location for marijuana cultivation because it is remote, primarily forested, and sparsely populated. Humboldt, Mendocino, and Trinity Counties, the three major counties known for marijuana cultivation in Northwestern California [5], comprise 7% (26,557 km^2^) of the total land area of the state of California. However, their combined population of 235,781 accounts for only 0.62% of the state’s total population (United States Census Data 2012). Humboldt County, with an area of 10,495 km^2^, has over 7689 km^2^ of forestland comprising more than 70% of its land base. More importantly, Humboldt County has 5,317 km^2^ of private lands on over 8,000 parcels zoned for timber production [6]. This makes Humboldt County a feasible place to purchase small remote parcels of forestland for marijuana cultivation.

The broad array of impacts from marijuana cultivation on aquatic and terrestrial wildlife in California has only recently been documented by law enforcement, wildlife agencies, and researchers. These impacts include loss and fragmentation of sensitive habitats via illegal land clearing and logging; grading and burying of streams; delivery of sediment, nutrients, petroleum products, and pesticides into streams; surface water diversions for irrigation resulting in reduced flows and completely dewatered streams [2,7–10]; and mortality of terrestrial wildlife by rodenticide ingestion [11,12]. Though these impacts have been documented by state and federal agencies, the extent to which they affect sensitive fish and wildlife species and their habitat has not been quantified. These impacts have gained attention in recent years [7,9] because of the continuing prevalence of “trespass grows,” illicit marijuana cultivation on public land. In comparison, the extent of cultivation and any associated environmental impacts on private lands are poorly understood, primarily because of limited access. In addition, state and local agencies lack the resources to address environmental impacts related to cultivation on private lands. In contrast with many MCSs on public lands, MCSs on private lands appear to be legal under state law, pursuant to Proposition 215. Regardless of the legal status of these MCSs, the water use associated with them has become an increasing concern for resource agencies [13].

California’s Mediterranean climate provides negligible precipitation during the May—September growing season. In Northern California, 90–95% of precipitation falls between October and April [14]. Marijuana is a high water-use plant [2,15], consuming up to 22.7 liters of water per day. In comparison, the widely cultivated wine grape, also grown throughout much of Northwestern California, uses approximately 12.64 liters of water per day [16]. Given the lack of precipitation during the growing season, marijuana cultivation generally requires a substantial amount of irrigation water. Consequently, MCSs are often situated on land with reliable year-round surface water sources to provide for irrigation throughout the hot, dry summer growing season [7,8,12]. Diverting springs and headwater streams are some of the most common means for MCSs to acquire irrigation water, though the authors have also documented the use of groundwater wells and importing water by truck.

The impacts to aquatic ecosystems from large hydroelectric projects and other alterations of natural flow regimes have been well documented [17–20], but few studies have attempted to quantify the impacts of low-volume surface water diversions on stream flows [21,22]. A study in the Russian River watershed in Sonoma County, CA, concluded that the demand of registered water diversions exceeded stream flows during certain periods of the year, though this study did not quantify unregistered diversions. In addition, this study indicates that these registered diversions have the potential to depress spring base flows and accelerate summer recession of flows [22]. We postulate that the widespread, increasing, and largely unregulated water demands for marijuana cultivation, in addition to existing domestic demands, are cumulatively considerable in many rural Northern California watersheds.

In northern California, unregulated marijuana cultivation often occurs in close proximity to habitat for sensitive aquatic species. Because of this proximity and the water demands associated with cultivation, we chose to focus on the cumulative impacts of low-volume surface water diversions associated with marijuana cultivation. We evaluate these water demands at a watershed scale to determine whether they could have substantial effects on streamflow during the summer low-flow period. In addition, we discuss which sensitive aquatic species are most likely to be impacted by stream diversions and describe the nature of these impacts.

## Methods

Methods are presented for the following components of the study: study area selection, data collection, water use estimates, and hydrologic analysis. For the purposes of this study, a MCS is defined as any area where marijuana is grown, either outdoors or inside a greenhouse, based on our aerial image interpretation. Because marijuana cultivation is federally illegal, its scope and magnitude are difficult to measure precisely [2,4,23]. However, the authors have accompanied law enforcement on search warrants and site inspections to evaluate more than 40 MCSs in the Eel River watershed and other watersheds in northwestern California. During these site inspections the number, size, and arrangement of marijuana plants were recorded, as were the water sources, conveyance and storage methods. These on-the-ground verification data were used as the basis for identifying characteristics of MCSs from aerial images.

### Study Areas

**Fig 1:**
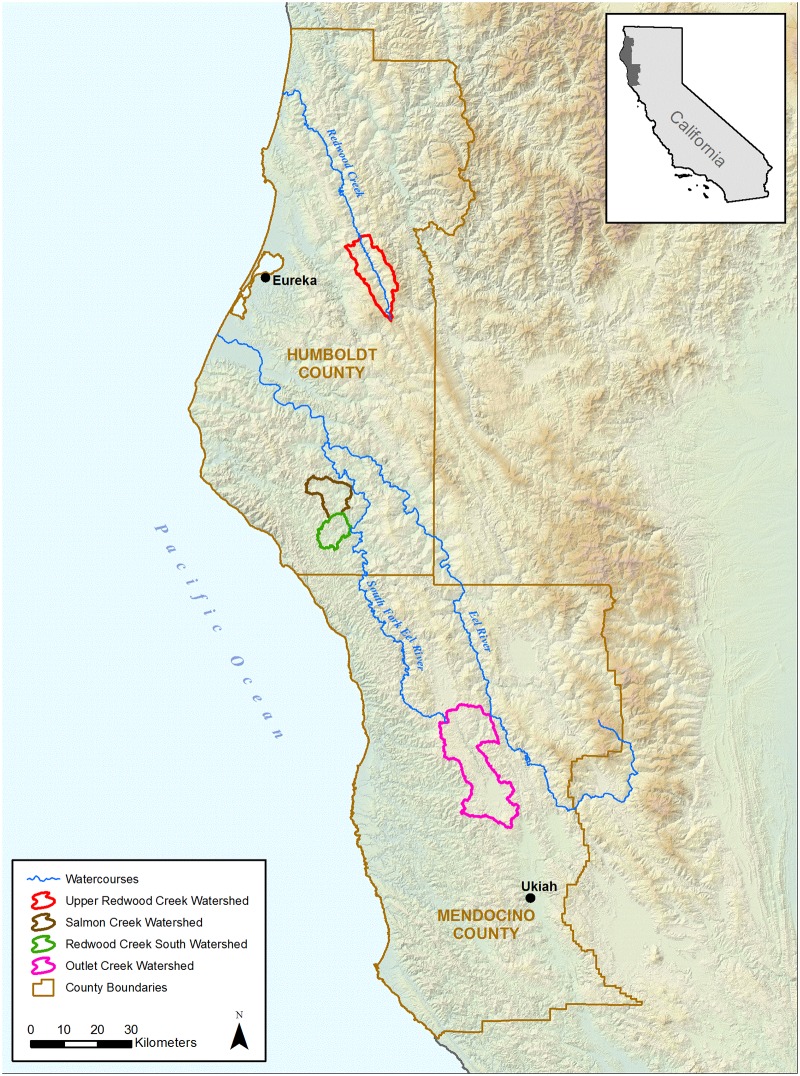
Study Watersheds and Major Watercourses.

**Fig 2:**
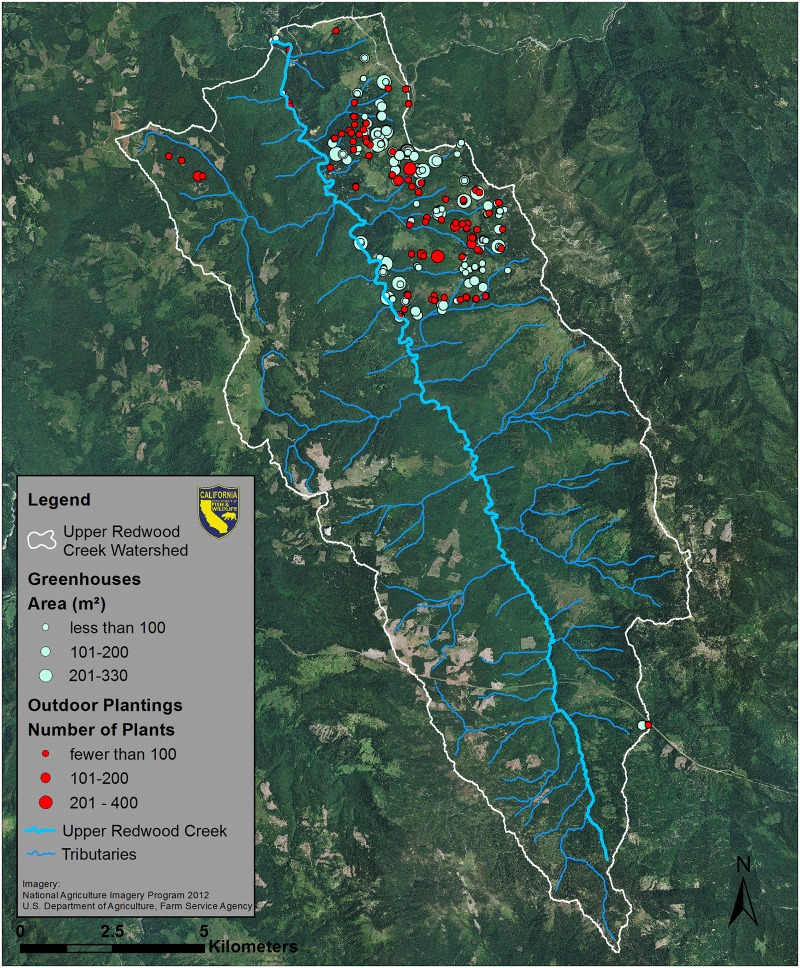
Upper Redwood Creek Watershed. Outdoor marijuana plantings are marked in red and greenhouses are marked in light green.

**Fig 3:**
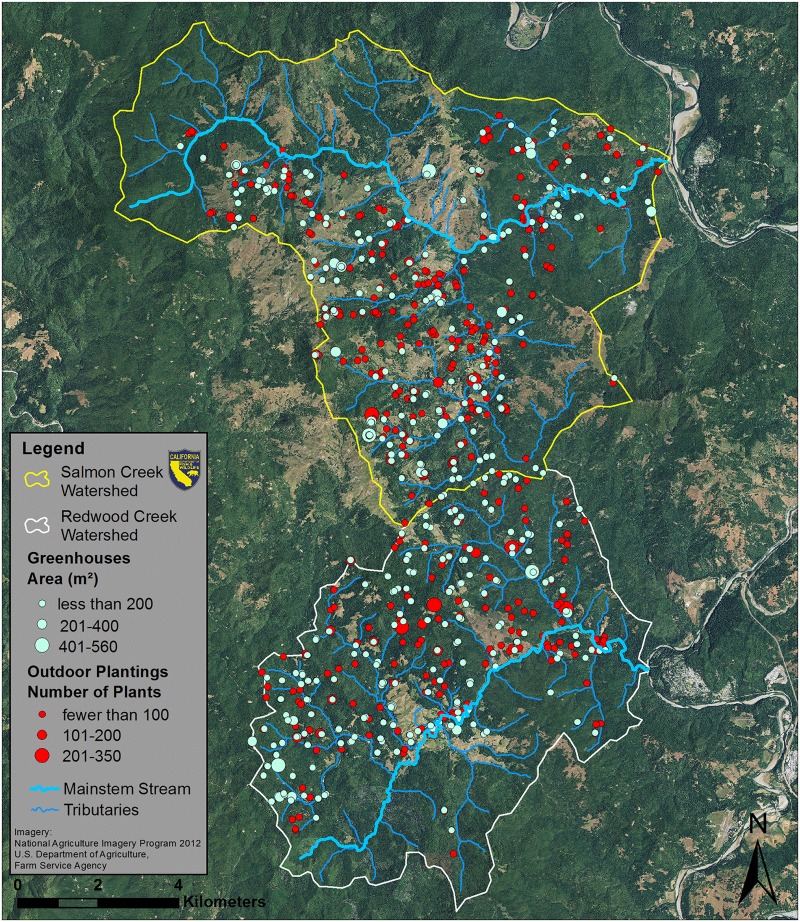
Salmon Creek and Redwood Creek South Watersheds. Outdoor marijuana plantings are marked in red and greenhouses are marked in light green.

**Fig 4:**
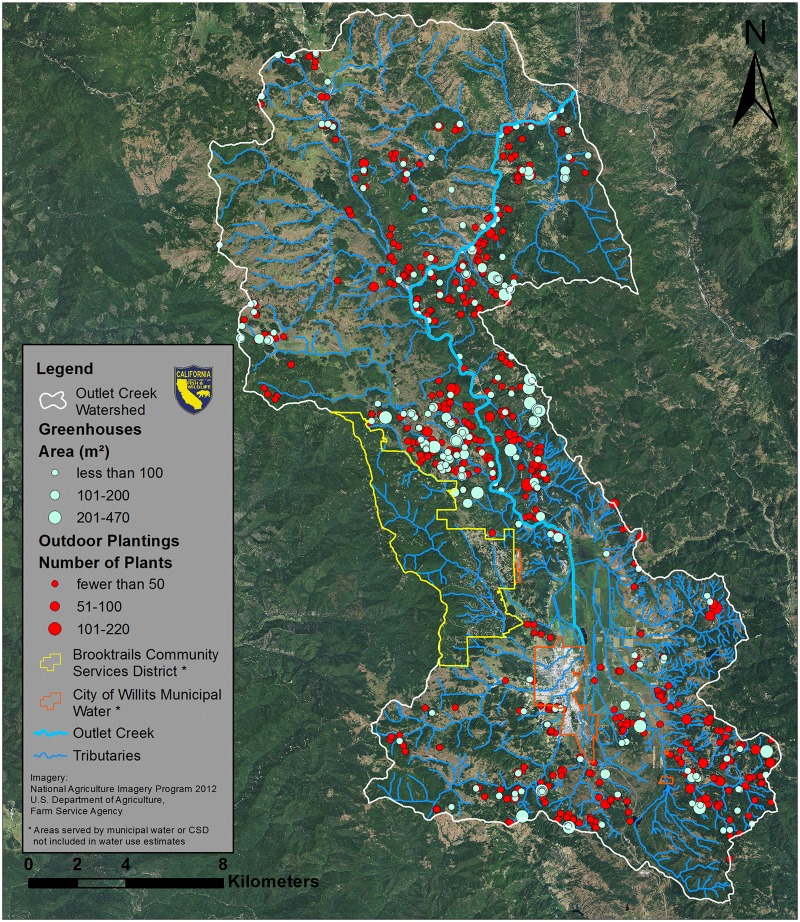
Outlet Creek Watershed. Outdoor marijuana plantings are marked in red and greenhouses are marked in light green.

Four study watersheds were selected—Upper Redwood Creek, Salmon Creek, and Redwood Creek South, located in Humboldt County; and Outlet Creek, located in Mendocino County (Figs. 1–4). Study watersheds were selected using the following criteria: (1) they are dominated by privately owned forestlands and marijuana cultivation is widespread within their boundaries as verified by low altitude survey flights and aerial imagery. (2) The primary watercourse, or downstream receiving body, has documented populations of sensitive aquatic species, such as coho salmon (*Oncorhynchus kisutch*). (3) Watersheds are of sufficient size so as to allow realistic population-scale and regional ecological relevance, but are not so large that conducting an analysis would be infeasible given limited staffing resources. (4) Streams in the watershed had either a flow gage, or nearby streams were gaged, which would allow proxy modeling of the low-flow period in the study watershed.

### Habitat

The study watersheds are dominated by a matrix of open to closed-canopy mixed evergreen and mixed conifer forests with occasional grassland openings. Dominant forest stands include Tanoak (*Notholithocarpus densiflorus*) and Douglas-fir (*Pseudotsuga menziesii*) Forest Alliances (“Alliance” is a vegetation classification unit that identifies one or more diagnostic species in the upper canopy layer that are indicative of habitat conditions) [24]. These forests are dominated by Douglas—fir, tanoak, madrone (*Arbutus menziesii*), big leaf maple (*Acer macrophyllum*), and various oak species (*Quercus* spp.). The Redwood (*Sequoia sempervirens*) Forest Alliance, as described by Sawyer et al. [24] is dominant in areas of Upper Redwood Creek and in lower Salmon Creek and Redwood Creek South and includes many of the same dominant or subdominant species in the Tanoak and Douglas-fir Forest Alliances. These watersheds, a product of recent and on-going seismic uplift, are characterized as steep mountainous terrain dissected by an extensive dendritic stream pattern, with the exception of Upper Redwood Creek, which has a linear trellised stream pattern [25].

### Data Collection and Mapping Overview

Study watershed boundaries were modified from the Calwater 2.2.1 watershed map [26] using United States Geological Survey (USGS) 7.5 minute Digital Raster Graphic images to correct for hydrological inconsistencies. These watershed boundaries and a reference grid with one square kilometer (km^2^) cells were used in Google Earth mapping program and ArcGIS (version 10.x, ESRI, Redlands, CA). Using Google Earth’s high-resolution images of northern California (image dates: 8/17/11, 7/9/12, and 8/23/12) as a reference, features of interest such as greenhouses and marijuana plants were mapped as points in ArcGIS. We identified greenhouses by color, transparency, elongated shape, and/or visible plastic or metal framework. Although we could not confirm the contents of greenhouses, the greenhouses we measured were generally associated with recent land clearing and other development associated with the cultivation of marijuana, as observed in our site inspections with law enforcement. Greenhouses clearly associated with only non-marijuana crop types, such as those in established farms with row crops, were excluded from our analysis. We identified outdoor marijuana plants by their shape, color, size and placement in rows or other regularly spaced configurations. We measured greenhouse lengths and widths using the Google Earth “Ruler” tool to obtain area, and counted and recorded the number of outdoor marijuana plants visible within each MCS. We also examined imagery from previous years using the Google Earth “Historical Imagery” tool to confirm that outdoor plants were not perennial crops, such as orchards.

### Plant Abundance and Water Use Estimates

For each watershed, we totaled the number of marijuana plants that were grown outdoors and combined this value with an estimated number of marijuana plants in greenhouses to get a total number of plants per watershed. To develop a basis for estimating the number of marijuana plants in greenhouses, we quantified the spatial arrangement and area of marijuana plants in 32 greenhouses at eight different locations in four watersheds in Humboldt County while accompanying law enforcement in 2013. We calculated 1.115 square meters (m^2^) per plant as an average spacing of marijuana plants contained within greenhouses. For the purposes of this study, we assume that the average greenhouse area to plant ratio observed by the authors on law enforcement visits was representative of the average spacing used at MCSs in the study watersheds.

Our water demand estimates were based on calculations from the 2010 Humboldt County Outdoor Medical Cannabis Ordinance draft [27], which states that marijuana plants use an average of 22.7 liters per plant per day during the growing season, which typically extends from June-October (150 days). Water use data for marijuana cultivation are virtually nonexistent in the published literature, and both published and unpublished sources for this information vary greatly, from as low as 3.8 liters up to 56.8 liters per plant per day [7,28]. The 22.7 liter figure falls near the middle of this range, and was based on the soaker hose and emitter line watering methods used almost exclusively by the MCSs we have observed. Because these water demand estimates were used to evaluate impacts of surface water diversion from streams, we also excluded plants and greenhouses in areas served by municipal water districts (Outlet Creek, Fig. 4).

### Hydrologic Analyses: Estimating Impacts on Summer Low Flows

**Fig 5:**
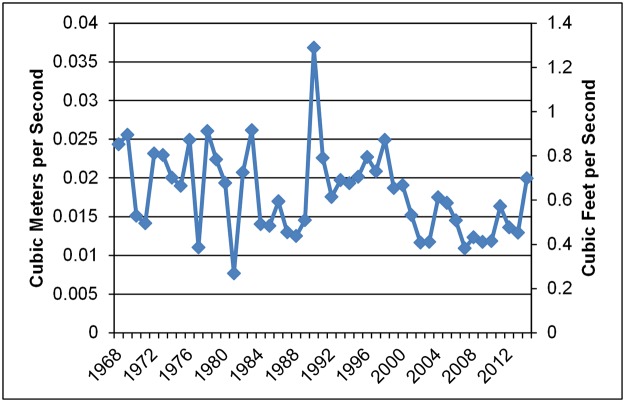
Elder Creek annual seven-day low flow. Values are shown for the period of record (water years 1968–2014).

The annual seven-day low flow, a metric often used to define the low flow of a stream, is defined as the lowest value of mean discharge computed over any seven consecutive days within a water year. This value varies from year to year. Annual seven-day low flow values for the ungaged watersheds in this study were estimated by correlating to nearby USGS gaged streams. Annual seven-day low flow values for Elder Creek (Fig. 5), a gage used for this correlation, demonstrate the year-to-year variability in the study watersheds. Elder Creek is considered to be the least disturbed of the gaged watersheds, and is also the smallest, with a contributing area of 16.8 square kilometers. The annual seven-day low flow estimates were made by scaling the gaged data by the ratio of average flow of the ungaged and gaged stream, a method that provides better estimates than scaling by watershed area [29]. Regression equations based on average annual precipitation and evapotranspiration were used to estimate average annual flow, providing a more unique flow characterization than using watershed area alone. These methods were developed by Rantz [30]. The gaged data were either from within the watershed of the study area or from a nearby watershed. Correlation with daily average flow data from a gaged stream makes sense when the ungaged watershed is considered to be hydrologically similar to the gaged watershed, i.e. similar geology, vegetation, watershed size and orientation, and atmospheric conditions (precipitation, cloud cover, temperature). The accuracy of gaged data at low flows can be problematic because gaging very low flows is difficult and limited depending on the location of the gage and the precision in low-flow conditions, but the method can still provide a rough estimate of low flow by taking into account the range of uncertainty. Data were used from the closest most relevant gaged watershed for correlation to the ungaged sites.

Data for the gaged stations are shown in Table 1. This table includes the estimated average annual flow calculated from both the gaged data and also by use of the regression equations for comparison. The annual seven-day low flow for the period of record of each of the gaged stations is shown in Table 2. This table also shows the minimum, average, and maximum seven-day low flow values over the period of record as a way to represent the variability of the low flow from year to year. To estimate the annual seven-day low flow for the ungaged streams, the average annual seven-day low flow of the gaged stream was multiplied by the ratio of the annual average streamflow of the ungaged stream and the annual average streamflow of the gaged stream. A range of values, including the lowest and highest estimate for each location were calculated to represent the annual variability.

**Table 1:** USGS stream gages in or near study watersheds.

Watershed	Gage	Period of Record	Area (km^2^)	MAP^a^ (cm/yr)	PET^b^ (cm/yr)	Mean Annual Runoff (cm/yr)	Q^c^avg (CMS^d^), predicted	Qavg (CMS), gaged	% difference
South Fork Eel River	USGS 11476500	10/1/1930–9/30/2012	1390.8	192.8	101.6	129.0	57.8	52.0	-11.1
Bull Creek	USGS 11476600	10/1/1967–9/30/2012	72.5	166.4	101.6	102.6	2.4	3.3	27.1
Elder Creek	USGS 11475560	10/1/1967–9/30/2012	16.8	215.9	101.6	152.1	0.8	0.7	-14.9
Outlet Creek	USGS 11472200	10/1/1956–9/30/1994	417.0	152.9	101.6	89.2	12.1	11.1	-8.8
Upper Redwood Creek	USGS 11481500	10/01/1953–10/1/2013	175.3	231.1	86.4	173.5	9.6	8.5	-12.6
Redwood Creek South	Ungaged	N/A	64.7	157.2	101.6	93.5	0.46	N/A	N/A
Salmon Creek	Ungaged	N/A	95.1	151.4	101.6	87.6	0.48	N/A	N/A

^a^mean annual precipitation

^b^potential evapotranspiration

^c^flow

^d^cubic meters per second

**Table 2:** Annual seven-day low flow range for period of record.

Gage	Seven-day low flow for period of record in cubic meters per second		
	Minimum	Average	Maximum
SF Eel Miranda	0.3519	0.8829	1.796
Bull	0.0059	0.0310	0.0853
Elder	0.0076	0.0180	0.0368
Outlet Creek	0.0000	0.0162	0.0498
Upper Redwood Creek	0.0265	0.1064	0.2601
Redwood Creek South (based on Elder Creek)	0.004	0.010	0.021
Salmon Creek (based on Elder Creek)	0.005	0.011	0.022

The mean annual streamflow of each ungaged stream was estimated using a regression equation, based on estimates of runoff and basin area developed by Rantz [30] (Equation 1). The mean annual runoff was estimated from a second regression equation (Equation 2) based on the relationship between mean annual precipitation and annual potential evapotranspiration for the California northern coastal area [30]. Mean annual precipitation values are from the USGS StreamStat web site (http://water.usgs.gov/osw/streamstats/california.html), which uses the PRISM average area weighted estimates based on data from 1971–2000. The estimates of mean annual evapotranspiration were taken from a chart produced by Kohler [31].

$${\text{Q}}_{\text{Avg}}=\;0.07362=\left(\frac{\text{m}³}{\text{sec}}\times \text{yr}\times \text{cm}\times \text{km}²\right)\times \text{R}\times \text{A}$$ eq. (1)

With

R=MAP−0.4  (PET)−9.1 eq. (2)

Where

$${\text{Q}}_{\text{Avg}}\;=mean\;annual\;discharge\;\left(\frac{\text{m}³}{\text{sec}}\right)$$

$$\text{R }=\;mean\;annual\;runoff\;\left(\frac{\text{cm}}{\text{yr}}\right)\text{​}$$

A   = drainage area (km²)

$$\text{MAP }=\;mean\;annual\;precipitation\;\left(\frac{\text{cm}}{\text{yr}}\right)$$

$$\text{PET}\;=\;potential\;evapotranspiration\;\left(\frac{\text{cm}}{\text{yr}}\right)$$

Estimates of average annual flow made by using these equations range from-15% to +27% below and above the calculated value using the gaged daily average data (Table 1). The Bull Creek gage estimate produced the largest deviation of 27% and may be considered an outlier because of the known disturbances in the watershed due to historic logging practices, and USGS reported “poor” low flow data.

**Fig 6:**
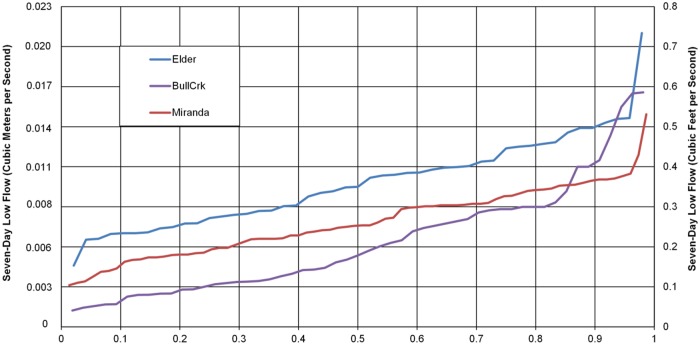
Duration curve of estimates of annual seven-day low flow for Redwood Creek South based on USGS data from nearby streams (Elder Creek, South Fork Eel at Miranda, and Bull Creek).

The mean annual flow for each ungaged watershed was calculated using the Rantz method described above. The mean annual precipitation and runoff values are shown in Table 1 with the predicted mean annual flow for the ungaged streams. The annual seven-day low flows for Upper Redwood Creek and Outlet Creek were calculated using data from their respective stream gages. For Redwood Creek South and Salmon Creek, both watersheds with no mainstem gage, the annual seven-day low flow was calculated in the same way by using the data from nearby gaged streams within the South Fork Eel watershed (Bull Creek, Elder Creek, and South Fork Eel near Miranda gage). Fig. 6 shows three different estimates of the duration curves of the annual seven-day low flow for the Redwood Creek South ungaged site based on the three different nearby gages. The variations between these estimated duration curves (Fig. 6) illustrate the relative variability of annual seven-day low flow. Reasons for this variability may include the difference in hydrologic response of the gaged watersheds from the ungaged watersheds, differences in withdrawals or low flow measurement error, differences in the atmospheric patterns over the watershed, or differences in watershed characteristics (watershed size, orientation, land use, slope etc.). The gaged watersheds differed from the study watersheds in several ways, such as size (Miranda gage), disturbance (Bull Creek gage), and distance and orientation from the study watersheds (Elder Creek gage). Despite the differences, the Elder Creek gage most likely represents the best data set for correlation to the ungaged watersheds based on its similar size and relative unimpairment. The estimated values represent the upper limit of low flows for the ungaged streams, thus are conservative values and may be an overestimate.

## Results

**Fig 7:**
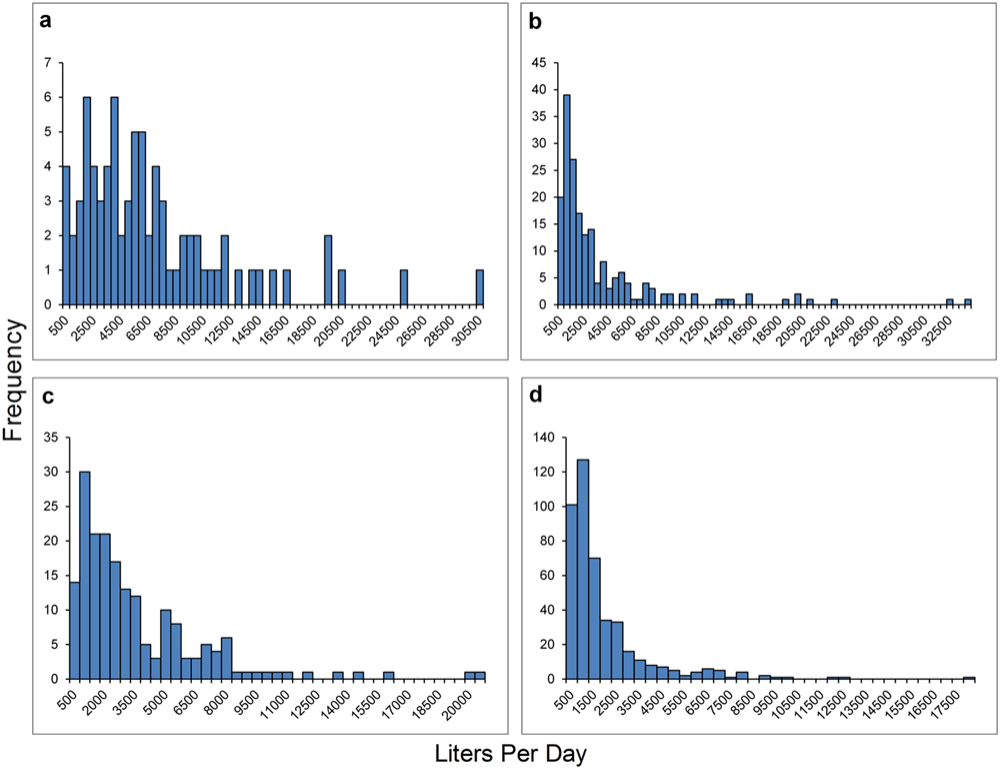
Frequency distribution of the water demand in liters per day (LPD) required per parcel for marijuana cultivation for each study watershed. (a) Upper Redwood Creek watershed, 79 parcels with marijuana cultivation, average water use 6622 LPD, (b) Salmon Creek watershed, 189 parcels with marijuana cultivation, average water use 3620 LPD, (c) Redwood Creek South watershed, 187 parcels with marijuana cultivation, average water use 3308 LPD, (d) Outlet Creek watershed, 441 parcels with marijuana cultivation, average 1642 LPD. See also S1 Table.

MCSs were widespread in all four study watersheds. In general, MCSs were clustered and were not evenly distributed throughout the study watersheds (Figs. 2–4). Estimated plant totals ranged from approximately 23,000 plants to approximately 32,000 plants per watershed (Table 3). Using the plant count estimates multiplied by our per plant daily water use estimate of 22.7 liters [27] we determined that water demands for marijuana cultivation range from 523,144 liters per day (LPD) to 724,016 LPD (Table 3). We also calculated the daily water use for each parcel that contained at least one marijuana cultivation site (S1 Table). Histograms showing the frequency distribution of daily water use per parcel are displayed for each watershed in Fig. 7. The majority of parcels in this study use an estimated 900 to 5,000 LPD for marijuana cultivation. These water use estimates are only based on irrigation needs for the marijuana plants counted or the greenhouses measured on that parcel, and do not account for indoor domestic water use, which in Northern California averages about 650 liters per day [32]. Thus, our water use demand estimates for marijuana cultivation are occurring in addition to domestic household uses that may occur and are also likely satisfied by surface water diversions.

**Table 3:** Marijuana mapping summary of four watersheds.

Watershed	Outdoor Plants	Green-houses (counted)	Total area, m^2^ (Green-houses)	Estimated Plants in Green-houses	Estimated Total Plants in Watershed	Estimated Water Use per Day (Liters)
Upper Redwood Creek	4,434	220	20749.4	18,612	23,046	523,144
Salmon Creek	11,697	302	20557.5	18,440	30,137	684,110
Redwood Creek South	10,475	324	18703.9	16,777	27,252	618,620
Outlet Creek	15,165	266	18651.1	16,730	31,895	724,016

Outdoor plants and greenhouses were identified from aerial images of Humboldt and Mendocino Counties. Greenhouse areas were estimated using the Google Earth measuring tool and an average area of 1.11484 m^2^ (converted from 12 ft^2^) per plant was used to estimate total number of plants in greenhouses.

Minimum and maximum annual seven-day low flow values in these watersheds (Table 2) ranged from 0.0–0.05 cubic meters per second (CMS) in Outlet Creek to. 03 -. 26 CMS in Upper Redwood Creek. By comparing daily water demands to minimum and maximum annual seven-day low flow values, we arrived at a range of values that represent water demand for marijuana cultivation as a percentage of stream flow in each watershed (Table 4, S2 Table). In Upper Redwood Creek, which had the greatest summer flows (Table 2), we estimate water demand for marijuana cultivation is the equivalent of 2–23% of the annual seven-day low flow, depending on the water year. In Redwood Creek South, our data indicate that estimated water demand for marijuana cultivation is 34–165% of the annual seven-day low flow, and in Salmon Creek, estimated water demand for marijuana is 36–173% of the annual seven-day low flow. In Outlet Creek, estimated demand was 17% of the maximum annual seven-day low flow. However, the percent of the annual seven-day low flow minimum could not be calculated because this minimum stream flow was undetectable at the gage (flow <0.00 CMS) in nine of 38 years during the period of record (1957–1994). Due to this minimum annual seven-day low flow of almost zero, marijuana water demand is greater than 100% of the minimum annual seven-day low flow, but we cannot determine by how much.

**Table 4:** Estimated water demand for marijuana cultivation expressed as a percentage of seven-day low flow in four study watersheds.

Watershed	Area (km^2^)	Plants per km^2^	Demand as percent of seven-day low flow	
			Percent of low flow maximum	Percent of low flow minimum
Upper Redwood Creek	175.3	131.6	2%	23%
Salmon Creek	95.1	316.9	36%	173%
Redwood Creek South	64.7	421.2	34%	165%
Outlet Creek	419.1	76.1	17%	>100%*

* The seven-day low flow minimum was measured as 0.0 CMS at the gage.

**Fig 8:**
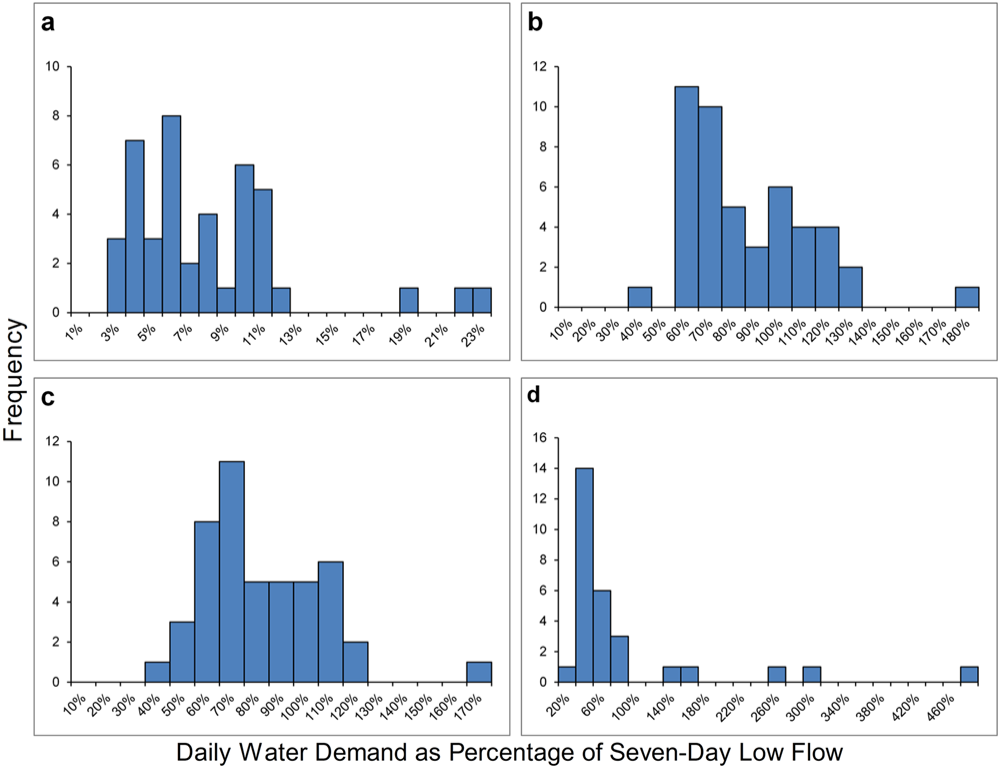
Frequency distribution of the water demand for marijuana cultivation as a percentage of seven-day low flow by year in each study watershed. Water demand data are from a remote sensing exercise using aerial imagery from 2011–2012 and are compared with each year’s annual seven-day low flow value for the period of record in each study watershed: (a) Upper Redwood Creek watershed (USGS gage near Blue Lake, CA, coverage from water year (WY) 1954–1958 and 1973–2014), (b) Salmon Creek watershed (data modeled using USGS gage on Elder Creek, CA, coverage from WY 1968–2014), (c) Redwood Creek South (data modeled using USGS gage on Elder Creek, CA, coverage from WY 1968–2014), and (d) Outlet Creek (USGS gage near Longvale, CA, coverage from WY 1957–1994). Data from WYs 1977, 1981, 1987–1989, and 1991–1994 are excluded from Outlet Creek watershed due to seven-day low flow values of zero at the gage. Water demand as a percentage of seven-day low flow would be >100% in these years, but we cannot determine by how much.

We also compared the per-watershed daily water demands to the seven-day low flow values for each year of data available in order to better understand the magnitude and frequency of these water demands (Fig. 8, S2 Table). Although substantial demand for water for marijuana cultivation is a more recent and growing phenomenon, by comparing the water use estimates from our remote sensing exercise to historical stream flow data we can better understand how this demand as a percentage of stream flow may vary over the years. Our results indicate that if the same level of water demand for marijuana cultivation had been present for the period of record of the gages, this demand would have accounted for over 50% of streamflow during the annual seven-day low flow period in the majority of years in the Redwood Creek South and Salmon Creek watersheds (based on Elder Creek gage data that spans from water year 1968–2014). In Outlet Creek, the annual seven-day low flow data varied greatly over the period of record (water year 1957–1994) and was too low to measure in nine of the 38 years. The seven-day low flow value was therefore recorded as zero, which means that the water demand was greater than 100% of streamflow, but we could not calculate the water demand as a percentage of stream flow in those years. In Upper Redwood Creek, water demand was much less pronounced in comparison to stream flow, with water demand never accounting for more than 23% of the annual seven-day low flow, and accounting for 10% or greater of the annual seven-day low flow in only 30% of years during the period of record (water year 1954–2014 with a gap between 1959–1972). To summarize, we estimate that in three of the four watersheds evaluated, water demands for marijuana cultivation exceed streamflow during low-flow periods.

## Discussion

### Aerial Imagery Limitations and Water Demand Assumptions

Due to a number of factors, it is likely that the plant counts resulting from aerial imagery interpretation (Table 3) are minimum values. The detection of marijuana plants using aerial imagery was found most effective for larger cultivation plots in forest clearings greater than 10 m^2^ because forest canopy cover and shadows can obscure individual plants or small plots, preventing detection. Some cultivators plant marijuana on a wide spacing in small forest canopy openings in order to avoid aerial detection [7,8]. The authors have also observed a variety of cultivation practices such as the use of large indoor cultivation facilities that could not be detected via aerial imagery. Moreover, a review of Google Earth historical aerial images after field inspections revealed that all MCSs visited in 2013 were either new or had expanded substantially since the previous year. Therefore, it is likely our results underestimate the total number of plants currently grown in these study watersheds and consequently underestimate the associated water demands.

Marijuana has been described as a high water-use plant [2,15] that thrives in nutrient rich moist soil [33]. Marijuana’s area of greatest naturalization in North America is in alluvial bottomlands of the Mississippi and Missouri River valleys where there is typically ample rain during the summer growing season [23,33]. Female inflorescences and intercalated bracts are the harvested portion of the marijuana plant. According to Cervantes [15], marijuana uses high levels of water for floral formation and withholding water stunts floral formation. Cervantes recommends marijuana plants be liberally watered and “allow for up to 10 percent runoff during each watering.”

There is uncertainty as to actual average water use of marijuana plants because there are few reliable published reports on marijuana water use requirements. As with the cultivation of any crop, variation in average daily water use would be expected based upon many variables, including the elevation, slope, and aspect of the cultivation site; microclimate and weather; size, age, and variety of the plant; native soil type and the amount and type of soil amendments used and their drainage and water retention characteristics; whether plants are grown outdoors, in greenhouses, or directly in the ground or in containers and the size of the container; and finally, the irrigation system used and how efficiently the system is used and maintained [34–36]. However, our water demand estimate of 22.7 L/day/plant based on the limited industry data available [27] comports with the U.S. Department of Justice 2007 Domestic Cannabis Cultivation Assessment [2], which indicates marijuana plants require up to 18.9 L/day/plant.

**Fig 9:**
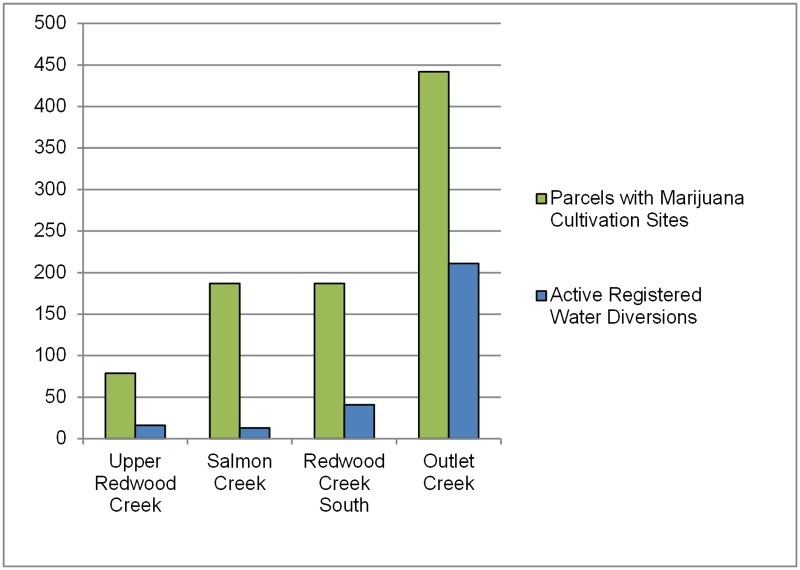
Active water rights in the study watersheds. Parcels with active registered water diversions (on file with California’s Division of Water Rights) compared to parcels with marijuana cultivation sites (MCSs) in the four study watersheds.

In many rural watersheds in Northern California, the primary source for domestic and agricultural water is from small surface water diversions [37]. These diversions must be registered with the State Water Resources Control Board (SWRCB), the agency responsible for administering water rights in California. SWRCB registrations are also subject to conditions set by the California Department of Fish and Wildlife in order to protect fish, wildlife, and their habitats. However, when querying the SWRCB’s public database, we found low numbers of registered, active water diversions on file relative to the number of MCSs we counted in the study watersheds. The total number of registered, active diversions on file with the SWRCB accounted less than half of the number of parcels with MCSs that were visible from aerial imagery (Fig. 9). In some watersheds, the number was as low as 6%. Since we do not know if the registered diversions on file with the SWRCB belong to parcels with MCSs, it is uncertain if the registered diversions in a particular watershed are connected with any of the MCSs we counted.

Our calculations of water demand as a percentage of stream flow assume that all potential water users are diverting surface water or hydrologically-connected subsurface flow. Historical water use practices and our field inspections with law enforcement support this assumption, although there are few hard data available as there are relatively few active registered water diversions on file with the Division of Water Rights when compared to the potential number of water users in the watersheds (Fig. 9).

Implicit in our calculations is the assumption that all water users are pumping water at the same rate throughout the day, as well as throughout the growing season. In reality, we expect water demand to gradually increase throughout the season as plants mature. This increased water demand would coincide with the natural hydrograph recession through the summer months, creating an even more pronounced impact during the summer low-flow period. In a similar study that monitored flow in relation to surface water abstraction for vineyard heat protection, flows receded abnormally during periods of high maximum daily temperature [21]. These results indicate that water users can have measureable effects on instantaneous flow in periods of high water demand. Our results suggest that similar impacts could occur during the summer low flow period in the study watersheds.

Additionally, our analysis assumes the water withdrawals will impact the entire watershed in an even, consistent way. In reality, we would expect water demand to be more concentrated at certain times of day and certain periods of the growing season, as described above. Furthermore, results of our spatial analysis indicate that MCSs are not evenly distributed on the landscape, thus impacts from water withdrawals are likely concentrated in certain areas within these watersheds. Because of these spatially and temporally clustered impacts, we may expect to see intensification of stream dewatering or temperature elevation in certain tributaries at certain times of year, which could have substantial impacts on sensitive aquatic species. Recent data indicate that peaks in high stream temperatures and annual low-flow events are increasing in synchrony in western North America [38], an effect that would be exacerbated by the surface water withdrawals we describe here. Further modeling and on-the-ground stream flow and temperature observations are needed to elucidate the potential extent of these impacts. The minimum streamflow estimates in Salmon Creek, Redwood Creek South, and Outlet Creek are so low that even a few standard-sized pumps operating at 38 liters per minute (LPM), which is a standard rate approved by the SWRCB for small diversions, could dewater the mainstem stream if more than four pumps ran simultaneously in any one area. It follows that impacts on smaller tributaries would be even more pronounced. In addition, on-site observations of MCS irrigation systems, though anecdotal, indicate many of these water conveyance, storage, and irrigation systems lose a substantial amount of water through leaks and inefficient design. This would significantly increase the amount of surface water diverted from streams beyond what would actually be needed to yield a crop. More study is needed to fully understand the impacts of MCS water demand on instantaneous flow in these watersheds.

Given that marijuana cultivation water demand could outstrip supply during the low flow period, and based on our MCS inspections and surface water diversion and irrigation system observations, we surmise that if a MCS has a perennial water supply, that supply would be used exclusively. However, for MCSs with on-site surface water sources that naturally run dry in summer, or are depleted though diversion, it is likely that direct surface water diversion is used until the source is exhausted, then water stored earlier in the year or imported by truck supplants the depleted surface water. It is difficult to determine to what degree imported water and wet season water storage is occurring. However, our on-site MCS inspections support the assumption that the vast majority of irrigation water used for marijuana cultivation in the study watersheds is obtained from on-site surface water sources and water storage and importation is ancillary to direct surface water diversions.

### Comparison of Water Demands to Summer Low Flows

Our results suggest that water demand for marijuana cultivation in three of the study watersheds could exceed what is naturally supplied by surface water alone. However, in Upper Redwood Creek, the data suggest that marijuana cultivation could have a smaller impact on streamflow, with demand taking up approximately 2% to 23% of flow (Table 4). This projected demand of flow contrasts with the 34% to >100% flow demand range in the other watersheds, most likely because Upper Redwood Creek has greater mean annual precipitation, less evapotranspiration, and generally higher stream flow than the other watersheds (Tables 1–2). Furthermore, approximately half of the Upper Redwood Creek watershed is comprised of either large timber company holdings or federal lands. As Fig. 2 illustrates, MCSs in Upper Redwood Creek are concentrated within a relatively small area of privately-owned land that has been subdivided. It stands to reason that if all the land within the Upper Redwood Creek watershed was subject to the subdivision and parcelization that has occurred in Redwood Creek South, Salmon Creek, or Outlet Creek, the potential impacts to stream flow would also be greater.

In Outlet Creek, our results indicate a large range of potential water demand as a percentage of streamflow, from 17% in a “wet” year to greater than 100% when the stream becomes intermittent, as it does during many summers. Our data indicate that impacts to streamflow will vary greatly depending on the individual watershed characteristics, whether the year is wetter or drier than average, and the land use practices taking place.

### Environmental Impacts

The extent of potential environmental impacts in these watersheds is especially troubling given the region is a recognized biodiversity hotspot. According to Ricketts et al. [39], the study watersheds occur within the Northern California Coastal Forests Terrestrial Ecoregion. This ecoregion has a biological distinctiveness ranking of “globally outstanding” and a conservation status of “critical” [39]. For example, Redwood National Park, 20 km downstream of the Upper Redwood Creek sub-basin, has approximately 100 km^2^ of old-growth redwood forest, which is one of the world’s largest remaining old-growth redwood stands. The study watersheds also occur within the Pacific Mid-Coastal Freshwater Ecoregion defined by Abell et al. [40]. This ecoregion has a “Continentally Outstanding” biological distinctiveness ranking, a current conservation status ranking of “Endangered” and its ranking is “Critical” with regards to expected future threats [40]. Not surprisingly, numerous sensitive species, including state- and federally-listed taxa, occur in the study watersheds or directly downstream (Table 5).

**Table 5:** Sensitive aquatic species with ranges that overlap the four study watersheds: Upper Redwood Creek (URC), Redwood Creek South (RCS), Salmon Creek (SC), and Outlet Creek (OC).

Scientific Name	Common Name	Conservation Status in California	Study Watershed
*Oncorhynchus kisutch*	coho salmon	State and federally-threatened	URC, RCS, SC, OC
*Oncorhynchus tshawytscha*	Chinook salmon	federally-threatened	URC, RCS, SC, OC
*Oncorhynchus clarki clarki*	coastal cutthroat trout	SSC^1^	URC
*Oncorhynchus mykiss*	steelhead trout	federally-threatened	URC, RCS, SC, OC
*Rana aurora*	northern red-legged frog	SSC	URC, RCS, SC, OC
*Rana boylii*	foothill yellow-legged frog	SSC	URC, RCS, SC, OC
*Rhyacotriton variegatus*	southern torrent salamander	SSC	URC, RCS, SC, OC
*Ascaphus truei*	coastal tailed frog	SSC	URC, RCS, SC
*Emys marmorata*	western pond turtle	SSC	RCS, SC, OC
*Margaritifera falcata*	western pearlshell	S1S2^2^	URC

^1^The California Department of Fish and Wildlife designates certain vertebrate species as Species of Special Concern (SSC) because declining population levels, limited ranges, and/or continuing threats have made them vulnerable to extinction. Though not listed pursuant to the Federal Endangered Species Act or the California Endangered Species Act, the goal of designating taxa as SSC is to halt or reverse these species’ decline by calling attention to their plight and addressing the issues of conservation concern early enough to secure their long-term viability.

^2^ The California Natural Diversity Database (CNDDB) designates conservation status rank based on a one to five scale, one being “Critically Imperiled”, five being “Secure”. Uncertainty about a rank is expressed by a range of values, thus a status of S1S2 indicates that there is uncertainty about whether *Margaritifera falcata* ranks as state “Critically Imperiled” (S1) or state “Imperiled” (S2) [41].

Our results indicate that the high water demand from marijuana cultivation in these watersheds could significantly impact aquatic- and riparian-dependent species. In the Pacific Coast Ecoregion, 60% of amphibian species, 16% of reptiles, 34% of birds, and 12% of mammals can be classified as riparian obligates, demonstrating the wide range of taxa that potentially would be affected by diminished stream flows [42]. The impacts of streamflow diversions and diminished or eliminated summer streamflow would however disproportionately affect aquatic species, especially those which are already sensitive and declining.

### Impacts to Fish

Northern California is home to some of the southernmost native populations of Pacific Coast salmon and trout (i.e., salmonids) and the study area is a stronghold and refugia for their diversity and survival. Every salmonid species in the study watersheds has some conservation status ranking (Table 5). California coho salmon, for example, have undergone at least a 70% decline in abundance since the 1960s, and are currently at 6 to 15% of their abundance during the 1940s [43]. Coho salmon populations in all four study watersheds are listed as threatened under both the California and the Federal Endangered Species Acts, and are designated as key populations to maintain or improve as part of the Recovery Strategy of California Coho Salmon [43].

Of California’s 129 native inland fish species, seven (5%) are extinct in the state or globally; 33 (26%) are in immediate danger of becoming extinct (endangered), and 34 (26%) are in decline but not at immediate risk of extinction (vulnerable) [44]. According to Katz et al. [45], if present population trends continue, 25 (78%) of California’s 32 native salmonid taxa will likely be extinct or extirpated within the next century.

The diminished flows presented by this study may be particularly damaging to salmonid fishes because they require clean, cold water and suitable flow regimes [44]. In fact, water diversions and altered or diminished in-stream flows due to land use practices have been identified as having a significant impact on coho salmon resulting in juvenile and adult mortality [43].

Additionally, all four study watersheds are already designated as impaired for elevated water temperature and sediment by the U.S. Environmental Protection Agency pursuant to the Clean Water Act Section 303(d). Reduced flow volume has a strong positive correlation with increased water temperature [44]. Increased water temperatures reduce growth rates in salmonids, increase predation risk [46], and increase susceptibility to disease. Warmer water also holds less dissolved oxygen, which can reduce survival in juvenile salmonids [44]. Both water temperature and dissolved oxygen are critically important for salmonid survival and habitat quality [47–50].

Reduced stream flows can also threaten salmonids by diminishing other water quality parameters, decreasing habitat availability, stranding fish, delaying migration, increasing intra and interspecific competition, decreasing food supply, and increasing the likelihood of predation [43]. These impacts can have lethal and sub-lethal effects. Experimental evidence in the study region suggests summer dry-season changes in streamflow can lead to substantial changes in individual growth rates of salmonids [51]. Complete dewatering of stream reaches would result in stranding and outright mortality of salmonids, which has been observed by the authors at a number of MCSs just downstream of their water diversions.

### Impacts to Amphibians

Water diversions and altered stream flows are also a significant threat to amphibians in the northwestern United States [52,53]. The southern torrent salamander (*Rhyacotriton variegatus*) and coastal tailed frog (*Ascaphus truei*) are particularly vulnerable to headwater stream diversions or dewatering, which could lead to mortality of these desiccation-intolerant species [54]. To maximize the compatibility of land use with amphibian conservation, Pilliod and Wind [53], recommend restoration of natural stream flows and use of alternative water sources in lieu of developing headwater springs and seeps.

Numerous studies have documented the extreme sensitivity of headwater stream-dwelling amphibians to changes in water temperature [55,56] as well as amounts of fine sediment and large woody debris [57,58]. Additionally, Kupferberg et al. and others [52,59] have demonstrated the impacts of altered flow regimes on river-dwelling amphibians. However, the threat of water diversion and hydromodification—or outright loss of flow—from headwaters streams has not been well-documented in the amphibian conservation literature. This is likely because illegal and unregulated headwater stream diversions did not exist at this scale until the recent expansion of marijuana cultivation in the region. In contrast, timber harvesting, which until recently was the primary land use in forested ecoregions in the western United States, does not typically divert headwater streams in the same manner as MCSs. Timber harvesting operations, at least in California, have state regulatory oversight that requires bypass flows to maintain habitat values for surface water diversions. Thus, the results of our study highlight an emerging threat to headwater amphibians not addressed in Lannoo [60], Wake and Vredenburg [61], or more recently in Clipp and Anderson [62]

### Future Water Demands and Climate Change

Flow modification is one of the greatest threats to aquatic biodiversity [63]. As in many parts of the world, the freshwater needed to sustain aquatic biodiversity and ecosystem health in our study area is also subject to severe competition for multiple human needs. The threats to human water security and river biodiversity are inextricably linked by increasing human demands for freshwater [64,65]. In California, irrigated agriculture is the single largest consumer of water, taking 70–80% of stored surface water and pumping great volumes of groundwater [44]. In our study area, agricultural demands account for 50–80% of all water withdrawals [66]. Only late in the last century have the impacts of water diversions on aquatic species become well recognized. However, these impacts are most often assessed on large regional scales, e.g. major rivers and alluvial valleys, and the large hydroelectric dams, reservoirs, and flood control and conveyance systems that regulate them [67].

Few studies thus far have assessed the impacts of many small agricultural diversions on zero to third order streams and their cumulative effects on a watershed scale [21,22]. On a localized scale, with regional implications, this study detects an emerging threat to not only aquatic biodiversity but also human water security, since surface water supplies most of the water for domestic uses in watersheds throughout Northwestern California [37]. In these watersheds, the concept of “peak renewable water,” where flow constraints limit total water availability [68], may have already arrived. In other words, the streams in the study watersheds simply cannot supply enough water to meet current demands for marijuana cultivation, other human needs, and the needs of fish and wildlife.

Due to climate change, water scarcity and habitat degradation in northern California is likely to worsen in the future. Regional climate change projections anticipate warmer average air temperatures, increases in prolonged heat waves, decreases in snow pack, earlier snow melt, a greater percentage of precipitation falling as rain rather than snow, a shift in spring and summer runoff to the winter months, and greater hydroclimatic variability and extremes [69–77]. Consequently, future hydrologic scenarios for California anticipate less water for ecosystem services, less reservoir capture, a diminished water supply for human uses, and greater conflict over the allocation of that diminished supply [70,71,75,78,79]. Climate change is expected to result in higher air and surface water temperatures in California’s streams and rivers in the coming decades, which in turn could significantly decrease suitable habitat for freshwater fishes [80–83]. Due to a warming climate, by 2090, 25 to 41% of currently suitable California streams may be too warm to support trout [84].

Already, gage data and climate stations in northwestern California show summer low flow has decreased and summer stream temperatures have increased in many of northern California’s coastal rivers, although these changes cannot yet be ascribed to climate change [85]. In an analysis of gage data from 21 river gaging stations, 10 of the gages showed an overall decrease in seven-day low flow over the period of record. This dataset included Upper Redwood Creek as well as the South Fork Eel River, the receiving water body for Redwood Creek South and Salmon Creek [85].

Our analysis suggests that for some smaller headwater tributaries, marijuana cultivation may be completely dewatering streams, and for the larger fish-bearing streams downslope, the flow diversions are substantial and likely contribute to accelerated summer intermittence and higher stream temperatures. Clearly, water demands for the existing level of marijuana cultivation in many northern California watersheds are unsustainable and are likely contributing to the decline of sensitive aquatic species in the region. Given the specter of climate change induced more severe and prolonged droughts and diminished summer stream flows in the region, continued diversions at a rate necessary to support the current scale of marijuana cultivation in northern California could be catastrophic for aquatic species.

Both monitoring and conservation measures are necessary to address environmental impacts from marijuana cultivation. State and federal agencies will need to develop more comprehensive guidelines for essential bypass flows in order to protect rearing habitat for listed salmonid species and other sensitive aquatic organisms. Installation of additional streamflow gages and other water quality and quantity monitoring will be necessary to fill data gaps in remote watersheds. In addition, increased oversight of water use for existing MCSs and increased enforcement by state and local agencies will be necessary to prevent and remediate illegal grading and forest conversions. Local and state governments will need to provide oversight to ensure that development related to MCSs is permitted and complies with environmental regulations and best management practices. Local and state agencies and nonprofit organizations should also continue to educate marijuana cultivators and the public about the environmental threats, appropriate mitigation measures, and permit requirements to legally develop MCSs and best protect fish and wildlife habitat. Finally, local governments should evaluate their land use planning policies and ordinances to prevent or minimize future forestland conversion to MCSs or other land uses that fragment forestlands and result in stream diversions.

## Supporting Information

S1 Table Number of outdoor plants counted, area of greenhouses measured, and estimated water use in Liters per day for each parcel in the study watersheds. (XLSX)

S2 Table Per-watershed daily water demands compared to seven-day low flow by year. (XLSX)
