# Correction: Impacts of Surface Water Diversions for Marijuana Cultivation on Aquatic Habitat in Four Northwestern California Watersheds

**DOI:** 10.1371/journal.pone.0137935

**Published:** 2015-09-03

**Authors:** Scott Bauer, Jennifer Olson, Adam Cockrill, Michael van Hattem, Linda Miller, Margaret Tauzer, Gordon Leppig

There are errors in the equations in the In the Hydrologic Analyses: Estimating Impacts on Summer Low Flows subsection of the Methods. These errors do not affect the results presented in the paper. Please view the complete, correct equation here:

$$Q_{Avg}=0.000317\times R\times A$$ Eq 1

with

R=MAP−0.4(PET)−23.11 Eq 2

where

$$Q_{Avg}=mean\;annual\;discharge\;\left(\frac{m^{3}}{\sec}\right)$$

$$R=mean\;annual\;runoff\;\left(\frac{cm}{yr}\right)$$

$$A=drainage\;area\;\left(km^{2}\right)$$

$$MAP=mean\;annual\;precipitation\;\left(\frac{cm}{yr}\right)$$

$$PET=potential\;evapotranspiration\;\left(\frac{cm}{yr}\right)$$
